# αCGRP-Induced Changes in Cerebral and Systemic Circulation; A TCD Study

**DOI:** 10.3389/fneur.2020.578103

**Published:** 2020-11-06

**Authors:** Darja Visočnik, Bojana Žvan, Marjan Zaletel, Matija Zupan

**Affiliations:** Department of Neurology, University Medical Center Ljubljana, University of Ljubljana, Ljubljana, Slovenia

**Keywords:** calcitonin gene-related peptide, migraine, cerebral arteries, transcranial doppler sonography, systemic hemodynamic

## Abstract

It is known that perivascular application of CGRP induces cerebral vasodilatation. However, it is unclear whether intravenous alfa CGRP (αCGRP) induces changes in cerebral and systemic hemodynamics. Therefore, we studied the influence of an αCGRP intravenous infusion at a rate of 1.5 mcg/min in 20 min on mean arterial velocity in the middle cerebral artery (vm MCA) and in the posterior cerebral artery (vm PCA) in twenty healthy subjects using transcranial Doppler (TCD). We found out that αCGRP decreased vm MCA (*p* < 0.001), vm PCA (*p* < 0.001), mean arterial pressure (MAP) (*p* < 0.001) and end-tidal CO_2_ (Et-CO_2_) (*p* = 0.030). The heart rate (HR) increased during αCGRP infusion (*p* < 0.001). In addition, we found a positive relationship between Et-CO_2_ and vm MCA (*p* = 0.001) as well as vm PCA (*p* = 0.043). In our view, αCGRP induces changes in cerebral and systemic circulation in healthy volunteers. It might cause vasodilatation of MCA and PCA and a compensatory decrease of Et-CO_2_ to αCGRP related hemodynamic changes.

## Introduction

Calcitonin gene-related peptide (CGRP) has been proven to play an important role in migraine and it has therefore become a target molecule in headache migraine research and treatment (1–5). There is growing evidence that alfa CGRP (αCGRP) importantly affects clinical and physiologic states.

Recently, it has been shown that patients with an excellent response to erenumab are highly susceptible to αCGRP provocation (6). Therefore, an αCGRP response should be regarded as an important predictive factor for selecting patients who are responders to anti-CGRP preventive treatment. In addition, current evidence suggests that CGRP is a potent dilatator of cerebral arteries (7–9). However, it is still unclear whether vasodilatation caused by αCGRP through intravascular interaction with an endogenous CGRP system, utilizes vascular endothelium.

Until now there have only been a few studies conducted on the effects of intravenous αCGRP on middle cerebral artery (MCA) dilatation and none on the response of the posterior cerebral artery (PCA) on CGRP. It has been shown that endothelium of anterior and posterior cerebral circulation could differ in function (10). Regarding cerebral effects of αCGRP, some studies demonstrated vasodilatation of MCA in response to intravenous αCGRP (7, 8), while others did not (9). In addition, the effect of αCGRP on systemic hemodynamics has been proven in animal and human studies. Earlier studies showed that αCGRP is a potent microvascular vasodilator but may not play an important role in the physiological control of systemic blood pressure (11). On the other hand, some studies have shown a significant decrease of systemic arterial pressure after receiving intravenous αCGRP (7). Thus, the cerebral and systemic effects of intravascular αCGRP are not completely understood. In addition, in the previous transcranial Doppler (TCD) studies in healthy volunteers for αCGRP effects on MCA mean velocity, a change in the CO_2_ concentration in exhaled air (Et-CO_2_) during intravenous αCGRP infusion was observed. The Et-CO_2_ significantly decreased during αCGRP application, but not when volunteers were treated with a placebo (7, 8). However, this phenomenon was not discussed and systematically studied. The polymodal monitoring of cerebral and systemic hemodynamic parameters is necessary to evaluate the hemodynamic effects of αCGRP stimulation. The aim of our study was to assess the influence of intravenous αCGRP on cerebral and systemic hemodynamic parameters using the TCD method. We hypothesize that intravenous αCGRP may have a significant vascular effect on both MCA and PCA and on systemic circulation.

## Materials and Methods

Twenty healthy subjects participated in our study (nine females aged 37.0 ± 2.8 years, 11 males aged 41.8 ± 7.6 years, *p* = 0.66). The inclusion criteria were age more than 18 years, normal somatic and neurological status, normal laboratory tests (complete blood count, serum potassium and sodium), and no atherosclerotic process of the carotid and vertebral arteries excluded by color-coded duplex sonography. The exclusion criteria were migraine and other primary headache disorders, previous cerebrovascular, endocrine, renal or liver diseases, uncontrolled hypertension, daily intake of medication except for contraceptives, pregnancy and breast-feeding.

The participants were free of tobacco, coffee, tea or any other food or beverages containing caffeine for at least 12 h before the start of the measurements. The color-coded duplex sonography of the carotid and vertebral arteries excluded an early atherosclerotic process in participants.

All participants were given written explanations about the experimental procedure and were informed that they were free to withdraw from the study at any time. They all gave written informed consent to participate in the study. The National Medical Ethics Committee of the Republic of Slovenia approved the study.

The experiments took place at 9:00 am in a quiet and dark room with constant temperature. During the experiment, the participants were resting in the supine position. Transcranial Doppler (TCD) sonography with 2 MHz ultrasound probes was applied to measure the mean flow velocity in MCA (vm MCA) through the left and mean flow velocity in PCA (vm PCA) through the right temporal acoustic window. The signals of the arteries were defined according to the direction of the blood flow, the typical depth of the signal and the response to compression. A mechanical probe holder was used to ensure a constant probe position. During the entire experiment the mean blood pressure (MAP) and heart rate (HR) were continuously measured using non-invasive plethysmography (Colin 7000, Komaki, Japan). The Et-CO_2_ was measured by an infrared capnograph (Capnograph, Model 9004, Smith medical, USA) using the standard protocol. The capnograph was connected to a breathing mask and to the computer. Et-CO_2_ signals were recorded on the same time scale as other variables. This enabled us to compare the signals and perform correlations between them.

The experiment consisted of a 10 min baseline period, a 20 min period during which an intravenous infusion of αCGRP 1.5 mcg/min (Calbiochem, Merck4Biosciences, Darmstadt, Germany) was given and a 10 min period after the end of the αCGRP infusion. TCD Multi-Dop X4 software (DWL, Sipplingen, Germany) was used to define average values of all parameters (vm MCA and vm PCA, MAP, HR and Et-CO_2_) during 5 min intervals. The first interval - point 0 was during the baseline period before starting αCGRP infusion (5–10 min of the experiment). The second interval - point 1 was 5–10 min after the start of αCGRP infusion (15–20 min of the experiment). The third interval - point 2 was 15–20 min after the start of αCGRP infusion (25–30 min of the experiment). The last interval - point 3 was 5–10 min after the end αCGRP infusion (35–40 min of the experiment). The mean vm MCA and vm PCA was calculated for each 5 min interval using the following equation vm∫= vdt/(t0–t5). The mean values of other variables (MAP, HR, Et-CO_2_) were also calculated for the same time intervals as the flow velocity using TCD software.

Sample size was driven from previous studies. For statistical analysis, SPSS version 21 was used. ANOVA for repeated measures was used to test differences among the successive points and a paired *t*-test was used to test the significance of differences between two points. Linear regression was used to test the correlations between the variables. All variables had value of the Shapiro-Wilk test >0.05.

## Results

**Figure 1:**
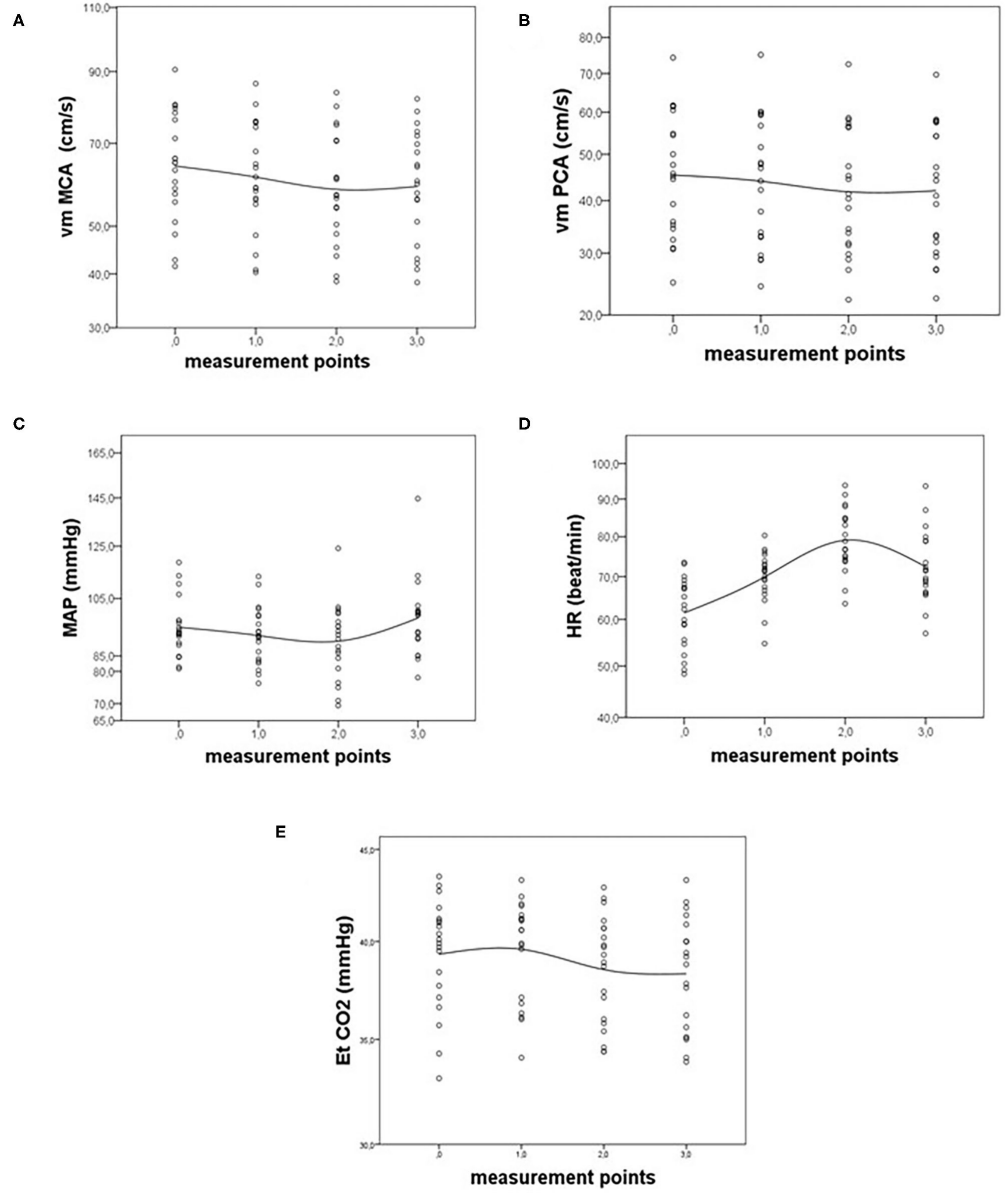
Statistical charts of **(A)** mean arterial velocity in middle cerebral artery (vm MCA), **(B)** mean arterial velocity in posterior cerebral artery (vm PCA), **(C)** mean arterial pressure (MAP), **(D)** heart rate (HR), and **(E)** end tidal carbon dioxide (Et-CO_2_) before (point 0), during (points 1 and 2), and after CGRP infusion (point 3).

First, we analyzed the vm MCA, vm PCA, MAP, HR, and Et-CO_2_ differences between the measurement points using the paired *t*-test. The statistical charts of the signals are presented in Figure 1. The probabilities (*p*-values) of differences between chosen points are shown in Table 1.

**Table 1:** The probability of differences (*p*-values) between measuring points using paired-*t*-test for vm MCA, vm PCA, MAP, HR, and Et-CO_2_.

**Pair points**	**vm MCA** **(*p*-value)**	**vm PCA** **(*p*-value)**	**MAP** **(*p*-value)**	**HR** **(*p*-value)**	**Et-CO**_**2**_ **(*p*-value)**
0–1	<0.001	0.003	0.062	<0.001	0.376
0–2	<0.001	<0.001	0.027	<0.001	0.023
0–3	<0.001	0.002	0.119	<0.001	0.066
1–2	<0.001	0.003	0.105	<0.001	0.004
1–3	0.062	0.020	0.001	0.075	0.016
2–3	0.401	0.710	<0.001	<0.001	0.463

*vm MCA, mean arterial velocity in middle cerebral artery; vm PCA, mean arterial velocity in posterior cerebral artery; MAP, mean arterial pressure; HR, heart rate; Et-CO_2_, end tidal carbon dioxide*.

The vm MCA showed a significant decrease in all points except between points 1–3 and 2–3 because of a slight increase of the signal at the recovery phase, after the end of the αCGRP infusion. The decrease of vm MCA was steady between points 1 and 2. The minimum value of vm MCA was measured at the point 2. The vm PCA changed in a similar way during the αCGRP infusion, except for vm PCA decreasing between points 1 and 3, which appeared to be significant in vm PCA but not in vm MCA (Figures 1A,B).

The MAP decreased during the αCGRP infusion and increased after it. The decrease was significant between measuring points 0 and 2, i.e., at the time of the CGRP infusion. The trends between points 0–1 and 1–2 were not significant. However, we observed a significant response of MAP between the points 1–3 and 2–3. There was no difference between points 0 and 3, which showed similar values for the MAP signal before and after the 10 min after the end of the αCGRP infusion (Figure 1C). HR significantly increased during the αCGRP infusion between points 0–1 and 1–2. After the end of the αCGRP infusion, the HR significantly decreased, which is represented by a significant difference between points 2 and 3. The final level of HR was similar to that in point 1 because we did not detect a significant difference between points 1 and 3 (Figure 1D).

The third part of our analysis was changes in Et-CO_2_ associated with the αCGRP infusion. The signal showed a remarkable and significant decrease during the αCGRP infusion between points 0–2 and 1–2. After the end of the infusion, there was no significant change of Et-CO_2_, which is between points 2 and 3 (Figure 1E).

Using linear regression, we analyzed correlations between vm MCA, vm PCA, MAP, HR and Et-CO_2_. We found significant positive correlations between vm MCA, vm PCA and Et-CO_2_. The correlation between vm MCA and vm PCA was significant. We did not find any significant correlations between mean velocity in both cerebral arteries and MAP and HR (Table 2). We found a significant negative correlation between Et-CO_2_ and HR as well as MAP. But we did not establish a relationship between MAP and HR. The results are presented in Table 2.

**Table 2:** Linear correlations between vm MCA, vm PCA, MAP, HR, and Et-CO_2_.

	**vm MCA**	**vm PCA**	**MAP**	**HR**	**Et-CO_**2**_**
vm MCA		Positive	Positive	Negative	Positive
		*p* <0.001	*p* = 0.744	*p* = 0.338	*p* = 0.001
		*r* = 0.645	*r* = 0.037	*r* = 0.109	*r* = 0.372
vm PCA	Positive		Positive	Positive	Positive
	*p* <0.001		*p* = 0.081	*p* = 0.281	*p* = 0.043
	*r* = 0.645		*r* = 0.197	*r* = 0.123	*r* = 0.227
MAP	Positive	Positive		Positive	Negative
	*p* = 0.744	*p* = 0.081		*p* = 0.111	*p* = 0.012
	*r* = 0.037	*r* = 0.197		*r* = 0.183	*r* = 0.280
HR	Negative	Positive	Positive		Negative
	*p* = 0.338	*p* = 0.281	*p* = 0.111		*p* = 0.007
	*r* = 0.109	*r* = 0.123	*r* = 0.183		*r* = 0.300
Et-CO_2_	Positive	Positive	Negative	Negative	
	*p* = 0.001	*p* = 0.043	*p* = 0.012	*p* = 0.007	
	*r* = 0.372	*r* = 0.227	*r* = 0.280	*r* = 0.300	

*The words positive or negative represent direction of correlations.*

*vm MCA, mean arterial velocity in middle cerebral artery; vm PCA, mean arterial velocity in posterior cerebral artery; MAP, mean arterial pressure; HR, heart rate; Et-CO_2_, end tidal carbon dioxide*.

## Discussion

The main finding of our study was a significant decrease of vm MCA and vm PCA during the αCGRP infusion at a rate of 1.5 mcg/min for 20 min (Figures 1A,B). The decrease of mean velocity in both arteries was steady through the entire αCGRP infusion with the lowest values just before the end of the infusion. After the end of the infusion, the velocities in both territories tended to increase, although insignificantly.

The arterial velocity reflects the relationship between blood flow and the cross-sectional area of the insonated artery. In the case where this relationship is constant and therefore the arterial velocity is constant, the dilatation of the artery due to CGRP effect should be followed by a proportional increase in cerebral blood flow (CBF). However, we found that during the αCGRP infusion, vm in MCA and PCA decreased. In addition, we found a highly positive correlation between vm MCA and vm PCA. Accordingly, the relationship between cerebral blood flow and arterial cross-sectional area also decreased. This could be due to proximal arterial vasodilatation or a decrease in CBF. In healthy volunteers, αCGRP increased global and regional CBF (8), but in migraine patients, there was no αCGRP effect on regional CBF. In our study, CBF was not measured because we wanted to evaluate an uncomplicated method for routine usage. Regardless of changes in CBF, we assume that the decrease in flow velocity induced by intravenous αCGRP might be evidence of MCA and PCA dilatation.

Our assumption is supported by results of extensive *in vitro* and *in vivo* research in animal and human studies that provide evidence that CGRP is a potent vasodilator and has greater potency compared to prostaglandin, acetylcholine and substance P (11). CGRP probably causes dilatation of cerebral arteries but has no effect on cerebral veins (12). Our results support the thesis that αCGRP induces dilatation of proximal cerebral arteries such as MCA and PCA. Indeed, previous studies showed that αCGRP is associated with MCA vasodilatation (7, 8). Lassen et al. found that αCGRP infusion dilates MCA in patients with migraines. The vm MCA significantly decreased during the αCGRP infusion at a rate of 2 mcg/min for 20 min in migraine patients compared to healthy subjects. The lowest value of vm MCA was measured 15 min after the start of the infusion, which was the last measurement during the αCGRP infusion. The regional CBF (rCBF) was measured using ^133^Xe SPECT at the baseline condition and after 15 min of the αCGRP infusion and it did not change during the αCGRP infusion. Petersen et al. in a study comparing efficacy of the CGRP receptor antagonist, BIBN4096BS, and a placebo in healthy volunteers, also found MCA dilatation caused by αCGRP infusion. In fact, their study was not set up to rule out αCGRP effects on MCA; however, the global CBF, rCBF and vm MCA was measured during and after the αCGRP infusion at a rate of 1.5 mcg/min for 20 min. The vm MCA was constant during the αCGRP infusion, but the global CBF and the rCBF increased after αCGRP administration. They calculated the relative percentage change of diameter of MCA and estimated that αCGRP increased the MCA diameter (8).

On the other hand, the study by Asghar did not show any change in the MCA circumference during an αCGRP infusion (9). In this study magnetic resonance angiography (MRA) was used to measure the changes of MCA circumference. The measurements were done before an αCGRP infusion and 10 min after the end of it. In our study, we found that after the end of the αCGRP infusion the velocities in MCA and PCA tended to increase, which could be evidence of arterial diameter normalization. Lassen also found no difference in vm MCA between the CGRP and the placebo group at the last measurement of vm MCA, 75 min after the start of the αCGRP infusion (7). The discrepancy between the Asghar study and our data could be due to the timing of MRA measurements. We suspect that the delay in MRA after the end of the CGRP stimulation attributed to their conclusions. Furthermore, the accuracy of MRA in the assessment of small but significant changes in MCA circumference is still not clarified (13).

At present, the mechanism of the αCGRP vasodilatation effect is only partly understood. Earlier studies in animals and *in vitro* in humans revealed that perivascular application of αCGRP induces dilatation of cerebral arteries (14). However, αCGRP given luminally failed to induce dilation of rat MCA (15). These findings are in accordance with the confirmed localization of CGRP receptors in arterial wall and support the opinion that CGRP-receptor components are present only in the cerebrovascular smooth muscle cells in arterial walls rather than in the cerebral endothelium. Furthermore, CGRP containing trigeminal nerves have been shown to surround the major cerebral and cortex pial arteries and that their stimulation releases CGRP, which in turn causes direct vasodilation via the receptors located on the vascular smooth muscle cells. On the other hand, it was confirmed that intravenous αCGRP causes dilatation of MCA and the middle meningeal artery as well (7–9). Furthermore, circulating CGRP receptor antagonists and antibodies against CGRP and the CGRP receptor, all of which are effective in migraine treatment, do not seem to be able to cross the blood-brain barrier to access targets in the cerebral vasculature (11). The results of our study speak in favor of an intravascular mechanism of αCGRP or effect at the regions outside the blood-brain barrier such as trigeminal ganglion. The cerebral arteries of migraineurs show enhanced response to exteroceptive stimuli probably mediated by CGRP (16). Therefore, we would expect more intensive response of arterial velocity and Et-CO_2_ to CGRP in a patient with migraine.

The next important finding of our study is that intravenous infusion of αCGRP at a rate of 1.5 mcg/min for 20 min significantly decreases MAP. The maximal decrease of MAP was at the end of the infusion. After that, a marked increase in MAP was observed. Changes in HR were also significant and in the opposite direction to the changes of MAP. The linear relationship between the variables was inconclusive. From this, we conclude that intravenous αCGRP decreases systemic blood pressure and causes an increase in HR, which is most probably due to cardiac output compensation of the decrease in MAP. Our results support the systemic effects of αCGRP in humans. In animal and human studies, intravenous administration of CGRP was associated with a decrease in systemic blood pressure and positive inotropic and chronotropic heart responses (11, 17). Furthermore, in our study no associations between MAP and vm MCA, as well as between MAP and vm PCA were found, which indicates uncoupling between cerebral flow and systemic arterial pressure and therefore normal regulation of CBF during and after αCGRP stimulation.

The interesting finding of our study was also that the Et-CO_2_ during the αCGRP infusion decreased significantly and persistently even after the end of the αCGRP infusion. The Et-CO_2_ decrease was reported in migraine patients during αCGRP infusion but not when treated with a placebo. The peak decrease in Et-CO_2_ occurred 5 min after the end of the αCGRP infusion (7). It seems that an Et-CO_2_ decrease does not occur incidentally. We also found positive and significant relationships between Et-CO_2_ and vm MCA as well as Et-CO_2_ and vm PCA. According to current knowledge, vm in MCA and PCA largely depends on cerebral arterioles status. Thus, αCGRP provokes important vasodilation at the arterioles' levels with the consequent putative increases in vm MCA and vm PCA. On the other hand, vasodilation/constriction of arterioles by CO_2_ concentration strongly determinates vm MCA and vm PCA. This is a well-known phenomenon named vasomotor reactivity to hyper/hypocapnia and it is one of the main control mechanisms to ensure constant cerebral perfusion (18, 19). Thus, depending on the stimulus, the vasodilatation could be largely distal, for example during pCO_2_ changes or largely proximal, for example the response to nitro-glycerine, or it could be distal and proximal (20). Therefore, we assume that a decrease in Et-CO_2_ during αCGRP stimulation tends to decrease CBF and consequently arterial velocities in MCA and PCA. The Et-CO_2_ decrease during the αCGRP stimulation could represent the compensation of an αCGRP-induced increase of cerebral blood flow in order to preserve intracranial pressure in the normal range. We could infer that Et-CO_2_ acts predominantly on distal resistant cerebral vessels, whereas αCGRP induces vasodilatation in proximal and distal parts of the cerebral arterial tree with a net decrease in arterial velocities in MCA and PCA. In other words, the relationship between blood flow and the cross-sectional area of the insonated artery decreased due to the vasodilatory properties of αCGRP on MCA and PCA. Moreover, we found significant negative relationships between Et-CO_2_ and MAP as well as Et-CO_2_ and HR. This could be explained by the common effect of αCGRP on Et-CO_2_, MAP and HR and not by causal relationships between the variables. Both Et-CO_2_ and HR seem to have a compensatory role in cerebral blood flow and systemic arterial pressure regulation, which have to be homeostatically preserved during an αCGRP infusion.

It is known that CGRP is derived predominantly from perivascular sensory neurons and the CGRP receptor complex was shown to be located on the smooth muscle cells of the cerebral vasculature. In addition, studies have shown that perivascular application of CGRP induced vasodilatation even after endothelial removal (21). The dilatory effect of CGRP on feline cerebral arteries is clearly endothelium independent, and the same is true for human cerebral, meningeal and temporal arteries (15). Furthermore, abluminal but not luminal application of αCGRP caused concentration-dependent vasodilatation of rat MCA. The results of these studies suggest the endothelial-independent action of CGRP. On the other hand, some studies confirm the endothelium-mediated vasodilatation of CGRP. In the periphery, CGRP and its receptors are present in endothelial cells and CGRP promotes the proliferation of endothelial cells (22). Human studies *in vivo* demonstrated the vasodilatation of cerebral arteries, and the medial meningeal and superficial temporal artery after intravenous infusion of αCGRP. Our study supports the intravascular effects of αCGRP probably via intravascular endothelium. In our study, we showed the effects of CGRP on cerebral and systemic circulation. Therefore, the potential long-term safety issues of CGRP antagonists could be raised in patients with coronary artery and cerebrovascular diseases and could be involved in guideline statements (23, 24).

The strength of the present study is the accurate description of the effects of CGRP in healthy human subjects. The potential limitations of the study are the low number of subjects and the lack of a control group.

## Conclusions

In conclusion, we found out that an intravenous αCGRP infusion might induce vasodilatation of MCA and PCA. It produces systemic effects with a significant decrease of MAP. The Et-CO_2_ decrease might have a compensatory role in preserving cerebral blood flow and intracranial pressure during CGRP stimulation. The αCGRP could be used as a marker for treatment efficacy of anti-CGRP monoclonal antibodies in migraine patients.

## Data Availability Statement

The raw data supporting the conclusions of this article will be made available by the authors, without undue reservation.

## Ethics Statement

The studies involving human participants were reviewed and approved by The National Medical Ethics Committee of the Republic of Slovenia. The participants provided their written informed consent to participate in this study.

## Author Contributions

BŽ, MZa, and DV contributed to conception and study design. DV organized database and managed experiments. MZa performed the statistical analyses and wrote the results section. DV wrote the first draft of the manuscript. All authors contributed to the article and approved the submitted version.

## Conflict of Interest

The authors declare that the research was conducted in the absence of any commercial or financial relationships that could be construed as a potential conflict of interest.
